# Ertugliflozin, renoprotection and potential confounding by muscle wasting. Reply to Groothof D, Post A, Gans ROB et al [letter]

**DOI:** 10.1007/s00125-021-05623-z

**Published:** 2022-03-03

**Authors:** David Z. I. Cherney, Bernard Charbonnel, Francesco Cosentino, Samuel Dagogo-Jack, Darren K. McGuire, Richard Pratley, Weichung J. Shih, Robert Frederich, Mario Maldonado, Annpey Pong, Christopher P. Cannon, on behalf of the VERTIS CV Investigators

**Affiliations:** 1 University of Toronto, Toronto, ON Canada; 2 University of Nantes, Nantes, France; 3 Unit of Cardiology, Karolinska Institute & Karolinska University Hospital, Stockholm, Sweden; 4 University of Tennessee Health Science Center, Memphis, TN USA; 5 University of Texas Southwestern Medical Center, Dallas, TX USA; 6 Parkland Health and Hospital System, Dallas, TX USA; 7 AdventHealth Translational Research Institute, Orlando, FL USA; 8 Rutgers School of Public Health, New Brunswick, NJ USA; 9 Rutgers Cancer Institute of New Jersey, New Brunswick, NJ USA; 10 Pfizer Inc., Collegeville, PA USA; 11 MSD Limited, London, UK; 12 Merck & Co., Inc., Kenilworth, NJ USA; 13 Cardiovascular Division, Brigham and Women’s Hospital, Harvard Medical School, Boston, MA USA

**Keywords:** Body weight, Chronic kidney disease, Ertugliflozin, Estimated glomerular filtration rate, Kidney outcomes, Sodium–glucose cotransporter 2 inhibitor

*To the Editor:* We wish to thank Groothof et al for their thoughtful letter regarding the relationship between muscle mass and kidney protection, especially changes in eGFR over time with the use of sodium–glucose cotransporter 2 (SGLT2) inhibitors [1]. As the authors indicate, SGLT2 inhibitors, including ertugliflozin, induce body weight loss of approximately 2–3 kg in most trials in people with type 2 diabetes mellitus with preserved kidney function. In their letter, Groothof et al raise the hypothesis that this body weight reduction is at least partly accounted for by a ‘substantial loss of muscle mass’, and that this may partly mimic kidney protection by mediating a decline in serum creatinine through decreased creatinine production. In their scenario, kidney protection is partly a biochemical epiphenomenon related to decreased creatinine production, rather than preservation of kidney function and solute clearance.

An important consideration against the hypothesis advanced by Groothof et al is the pattern of kidney function change compared with body weight change over time following SGLT2 inhibitor use. SGLT2 inhibitors induce an initial acute haemodynamic effect, characterised by a dip in eGFR, which is typically maximal by 4–8 weeks, followed by a return towards baseline by 12–16 weeks [2]. Body weight loss with SGLT2 inhibitors is quite rapid, with maximal effects being seen in the initial 4–8 weeks that then persist over time; these initial changes are, in large part, accounted for by reduced body water volume [3]. Accordingly, the temporal sequence of eGFR and body weight changes are dyssynchronous and are, therefore, unlikely to be physiologically related.

Beyond this chronological dissociation, existing data have not shown a consistent change in muscle mass in response to SGLT2 inhibition in either direction. In fact, in four [4–7] of the six trials [4–9] with dapagliflozin (which is pharmacologically similar to ertugliflozin) quoted by the authors, there was no effect on measures of lean mass [10], whilst a recent active comparator (glibenclamide) trial showed an increase in lean:total body mass ratio with dapagliflozin use [11]. In addition to the inconsistent directional effects on measures of lean body mass, we also wish to emphasise that, even if changes in lean body mass did occur, this variable is composed of several factors, including muscle and water content. Accordingly, in the unlikely scenario that lean mass was affected by the amounts suggested in several trials [10], at least 50% of a lean mass loss of generally <1.0 kg is accounted for by water loss [12]. From a quantitative perspective, this degree of muscle mass loss would not contribute to changes in eGFR in a clinically meaningful way over time.

A third set of observations that make it unlikely that body weight changes (as a surrogate for muscle mass) and kidney protection are related come from other trial cohorts [13–15]. We now know from dedicated trials in patients with kidney disease that SGLT2 inhibitors substantially reduce the risk of chronic kidney disease (CKD) progression, even in patients with baseline eGFR levels as low as 25 ml min^−1^ [1.73 m]^−2^, including those without type 2 diabetes [13, 14]. Importantly, in patients without diabetes and those with eGFR <30 ml min^−1^ [1.73 m]^−2^, the effects of SGLT2 inhibitors on glycaemic control and body weight are clinically negligible or neutral and, yet, profound kidney protection has been observed (not just based on eGFR decline but also on the number of events of end-stage kidney disease) [14, 15]. Importantly, this protection has been reported in heart failure (HF) trials, which also included individuals with CKD stage 4 and those without diabetes [16]. Although the eValuation of ERTugliflozin effIcacy and Safety CardioVascular outcomes (VERTIS CV) trial (ClinicalTrials.gov registration no. NCT01986881) did not enrol individuals with CKD stage 4 or necessarily include those at risk of CKD progression [17], as the authors point out, it is likely that the same principles apply in VERTIS CV [18].

Regarding the authors’ point that ‘therapy-related muscle wasting’ may conceal the need to start dialysis, changes in body weight and related muscle mass, if present at all, would occur very early following the commencement of SGLT2 inhibitor therapy, and there is no indication that body weight acts as a surrogate for muscle mass changes over time during the course of trials lasting 3–4 years [17]. Specifically, body weight loss plateaus early after initiation of SGLT2 inhibitor therapy and does not continue to decrease with chronic treatment [17]. Accordingly, previous body weight loss that is not progressive does not alter eGFR trends and, therefore, would not have an impact on decision making related to dialysis initiation.

Furthermore, the decision to initiate dialysis is not made based on biochemical factors (i.e. eGFR) alone, and would be made in conjunction with other clinical parameters, such as electrolyte levels and signs and/or symptoms of volume overload [19]. Hence, the authors’ suggestion that SGLT2 inhibitors may delay the start of dialysis by mitigating hypervolaemia is plausible, as these therapies reduce the risk of hospitalisation for HF across trials, including VERTIS CV [17], and avoidance of hypervolaemia may be an additional benefit in nephrology practice. Other methods to improve the operating characteristics of eGFR equations (including those using cystatin C) are welcome to better identify CKD and decide on its management, although existing data have shown that the effects of SGLT2 inhibitors on kidney function are consistent, regardless of the clearance method used [20–22].

## Data Availability

The data sharing policy, including restrictions, of Merck Sharp & Dohme Corp., a subsidiary of Merck & Co., Inc., (Kenilworth, NJ, USA), is available at http://engagezone.msd.com/ds_documentation.php. Requests for access to the clinical study data can be submitted through the EngageZone site or via email to: dataaccess@merck.com.

## Abbreviations

CKD: Chronic kidney disease
HF: Heart failure
SGLT2: Sodium–glucose cotransporter 2
VERTIS CV: eValuation of ERTugliflozin effIcacy and Safety CardioVascular outcomes

## References

[1] Groothof D, Post A, Gans ROB, Bakker SJL (2021) Ertugliflozin, renoprotection and potential confounding by muscle wasting. Diabetologia. 10.1007/s00125-021-05614-0 10.1007/s00125-021-05614-0 34940888

[2] Cherney DZI, Heerspink HJL, Frederich R (2020). Effects of ertugliflozin on renal function over 104 weeks of treatment: a post hoc analysis of two randomised controlled trials. Diabetologia.

[3] Lambers Heerspink HJ, de Zeeuw D, Wie L, Leslie B, List J (2013). Dapagliflozin a glucose-regulating drug with diuretic properties in subjects with type 2 diabetes. Diabetes Obes Metab.

[4] Kosugi R, Nakatani E, Okamoto K, Aoshima S, Arai H, Inoue T (2019). Effects of sodium-glucose cotransporter 2 inhibitor (dapagliflozin) on food intake and plasma fibroblast growth factor 21 levels in type 2 diabetes patients. Endocr J.

[5] Lundkvist P, Sjöström CD, Amini S, Pereira MJ, Johnsson E, Eriksson JW (2017). Dapagliflozin once-daily and exenatide once-weekly dual therapy: A 24-week randomized, placebo-controlled, phase II study examining effects on body weight and prediabetes in obese adults without diabetes. Diabetes Obes Metab.

[6] Sugiyama S, Jinnouchi H, Kurinami N et al (2018) Dapagliflozin reduces fat mass without affecting muscle mass in type 2 diabetes. J Atheroscler Thromb 25(6):467–476. 10.5551/jat.40873 10.5551/jat.40873 PMC6005223 29225209

[7] Tobita H, Sato S, Miyake T, Ishihara S, Kinoshita Y (2017) Effects of dapagliflozin on body composition and liver tests in patients with nonalcoholic steatohepatitis associated with type 2 diabetes mellitus: a prospective, open-label, uncontrolled study. Curr Ther Res Clin Exp 87:13–19. 10.1016/j.curtheres.2017.07.002 10.1016/j.curtheres.2017.07.002 PMC5587885 28912902

[8] Bolinder J, Ljunggren Ö, Kullberg J (2012). Effects of Dapagliflozin on Body Weight, Total Fat Mass, and Regional Adipose Tissue Distribution in Patients with Type 2 Diabetes Mellitus with Inadequate Glycemic Control on Metformin. J Clin Endocrinol Metab.

[9] Fadini GP, Bonora BM, Zatti G (2017). Effects of the SGLT2 inhibitor dapagliflozin on HDL cholesterol, particle size, and cholesterol efflux capacity in patients with type 2 diabetes: a randomized placebo-controlled trial. Cardiovasc Diabetol.

[10] Post A, Groothof D, Eisenga MF, Bakker SJL (2020) Sodium-glucose cotransporter 2 inhibitors and kidney outcomes: true renoprotection, loss of muscle mass or both? J Clin Med 9(5):1603. 10.3390/jcm9051603 10.3390/jcm9051603 PMC7291210 32466262

[11] Wolf VLW, Breder I, de Carvalho LSF (2021). Dapagliflozin increases the lean-to total mass ratio in type 2 diabetes mellitus. Nutr Diabetes.

[12] List JF, Woo V, Morales E, Tang W, Fiedorek FT (2009). Sodium-glucose cotransport inhibition with dapagliflozin in type 2 diabetes. Diabetes Care.

[13] Heerspink HJL, Jongs N, Chertow GM (2021). Effect of dapagliflozin on the rate of decline in kidney function in patients with chronic kidney disease with and without type 2 diabetes: a prespecified analysis from the DAPA-CKD trial. Lancet Diabetes Endocrinol.

[14] Chertow GM, Vart P, Jongs N et al (2021) Effects of dapagliflozin in stage 4 chronic kidney disease. J Am Soc Nephrol 32(9):2352–2361. 10.1681/ASN.2021020167 10.1681/ASN.2021020167 PMC8729835 34272327

[15] Heerspink HJL, Stefansson BV, Correa-Rotter R (2020). Dapagliflozin in patients with chronic kidney disease. N Engl J Med.

[16] Packer M, Anker SD, Butler J (2020). Cardiovascular and renal outcomes with empagliflozin in heart failure. N Engl J Med.

[17] Cannon CP, Pratley R, Dagogo-Jack S (2020). Cardiovascular outcomes with ertugliflozin in type 2 diabetes. N Engl J Med.

[18] Cherney DZI, Charbonnel B, Cosentino F (2021). Effects of ertugliflozin on kidney composite outcomes, renal function and albuminuria in patients with type 2 diabetes mellitus: an analysis from the randomised VERTIS CV trial. Diabetologia.

[19] Tattersall J, Dekker F, Heimbürger O (2011). When to start dialysis: updated guidance following publication of the Initiating Dialysis Early and Late (IDEAL) study. Nephrol Dial Transplant.

[20] Cherney DZI, Dekkers CCJ, Barbour SJ (2020). Effects of the SGLT2 inhibitor dapagliflozin on proteinuria in non-diabetic patients with chronic kidney disease (DIAMOND): a randomised, double-blind, crossover trial. Lancet Diabetes Endocrinol.

[21] van Ruiten CC, van der Aart-van der Beek AB, IJzerman RG (2021). Effect of exenatide twice daily and dapagliflozin, alone and in combination, on markers of kidney function in obese patients with type 2 diabetes: A prespecified secondary analysis of a randomized controlled clinical trial. Diabetes Obes Metab.

[22] Cherney DZ, Perkins BA, Soleymanlou N (2014). Renal hemodynamic effect of sodium-glucose cotransporter 2 inhibition in patients with type 1 diabetes mellitus. Circulation.

